# From (Cat) Scratch: A Unique Presentation of Central Nervous System Bartonella Infection

**DOI:** 10.7759/cureus.37044

**Published:** 2023-04-02

**Authors:** Wilson Rodriguez, Margarita Fedorova, Lokesh Rukmangadachar

**Affiliations:** 1 Neurology, Saint Louis University School of Medicine, Saint Louis, USA

**Keywords:** contrast enhancement, retinitis, immunosuppression, brain abscess, bartonella

## Abstract

Central nervous system manifestations of Bartonella species are rare and include meningitis, neuroretinitis, encephalitis, and isolated optic neuritis. We present the case of a 28-year-old woman who presented with a four-month history of progressive, asymmetric, bilateral painless vision loss. Her past medical history was significant for systemic lupus erythematosus. Notably, she had been on a high dose of prednisone for her immunosuppressive regimen. Brain MRI showed numerous contrast-enhancing lesions scattered throughout bilateral cerebral and cerebellar hemispheres and brainstem. She underwent a brain biopsy, and infection with Bartonella henselae was confirmed via a polymerase chain reaction. The patient was started on doxycycline and rifampin with improvement in vision and resolution of lesions as confirmed by a follow-up brain MRI. The literature review did not reveal any cases of multiple brain abscesses due to central nervous system Bartonella. Our case report aims to promote consider Bartonella infection as a cause of multiple brain abscesses in immunocompromised patients. It is essential to note that Bartonella can imitate other central nervous system infections, including toxoplasmosis, cryptococcosis, cysticercosis, and tuberculomas. Early identification is crucial as prompt treatment can lead to a complete cure.

## Introduction

The genus Bartonella encompasses a group of gram-negative pathogens that can infect erythrocytes, endothelial, and macrophage-derived cells [1,2]. Bartonella is often associated with cats, but it has been documented in a plethora of animals, including dogs or sand flies [3,4]. Central nervous system (CNS) manifestations of Bartonella species are rare and include meningitis, neuroretinitis, encephalitis, and isolated optic neuritis [4]. Diagnosis is usually performed with serology, Warthin-Starry silver staining, or confirmed with polymerase chain reaction (PCR). We describe a unique case of multiple necrotizing brain abscesses due to Bartonella henselae in an immunocompromised patient.

## Case presentation

A 28-year-old woman with a history of Systemic Lupus Erythematosus (SLE) and lupus nephritis on chronic immunosuppression presented to the Emergency Department (ED) with a four-month history of progressive, asymmetric, bilateral painless vision loss concerning for lupus retinitis.

**Figure 1:**
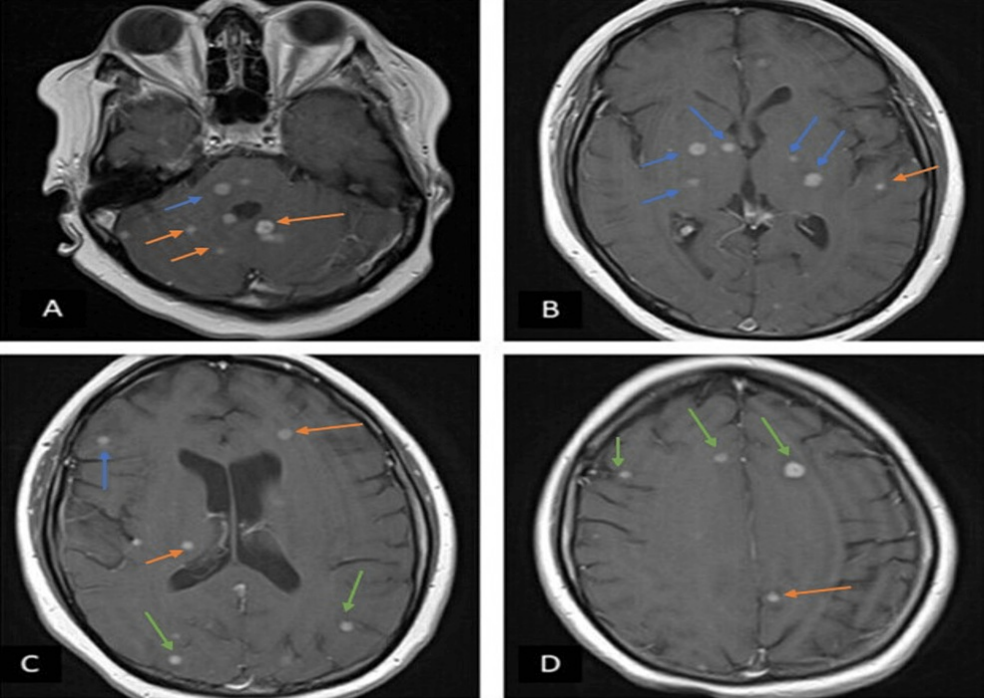
Initial brain MRI with contrast at admission (A) T1 with contrast axial sequence showing multiple small rounds enhancing lesions throughout pons (blue arrow) and bilateral cerebellar hemispheres (orange arrow). (B) T1 with contrast axial sequence showing multiple small rounds enhancing lesions throughout basal ganglia (blue arrow) and left temporal lobe (orange arrow). (C) T1 with contrast axial sequence showing multiple small rounds enhancing lesions in periventricular area (orange arrows), right subcortical frontal area (blue arrow) and bilateral subcortical parietal area (green arrows). (D) T1 with contrast axial sequence showing multiple small rounds enhancing lesions in bilateral frontal lobe (green arrows) and left parietal area (orange arrow).

Two months before presenting to the ED, she was evaluated by her ophthalmologist, who increased her prednisone regimen to 80 mg daily due to concern for lupus retinitis. On admission, she denied systemic symptoms. Her ophthalmologic exam was pertinent for decreased visual acuity (20/50 in the right eye and 20/400 in the left eye), chorio-retinal inflammatory lesions, and macular edema. No macular star was seen on the exam. Motor and sensory exams were unremarkable. MRI of the brain revealed numerous contrast-enhancing lesions throughout the brainstem, cerebral and cerebellar hemispheres (Figures 1A-1D).

Since the beginning, an infectious etiology was considered rather than SLE exacerbation given the multiple brain-enhancing lesions on the MRI, immunosuppression, history of immigration from Mexico, and exposure to cats at home. She was started on empiric therapy for CNS infection with ceftriaxone, ganciclovir, voriconazole, pyrimethamine, and sulfadiazine to cover potential opportunistic infections.

Infectious etiologies, including herpes simplex virus, Epstein-Barr virus, human immunodeficiency virus, mycobacteria, Histoplasma, Blastomyces, and Cryptococcus, were ruled out. Toxoplasma immunoglobulin G (IgG) was positive, and immunoglobulin M (IgM) was negative. Cerebrospinal fluid (CSF) analysis showed 1 WBC, 35 mg/dL protein, 76 mg/dL glucose, CSF PCR for Toxoplasma was negative, and flow cytometry with cytology was negative for CNS lymphoma. She was tested for Bartonella henselae and quintana; her IgM was negative; IgG titer was 1:64 (equivocal) and 1:256 (positive), respectively. The patient was continued on empiric treatment for cytomegalovirus CMV (20 days of ganciclovir followed by five days of valganciclovir) and Toxoplasma (six days of pyrimethamine with sulfadiazine) retinitis and brain abscess without improvement.

**Figure 2:**
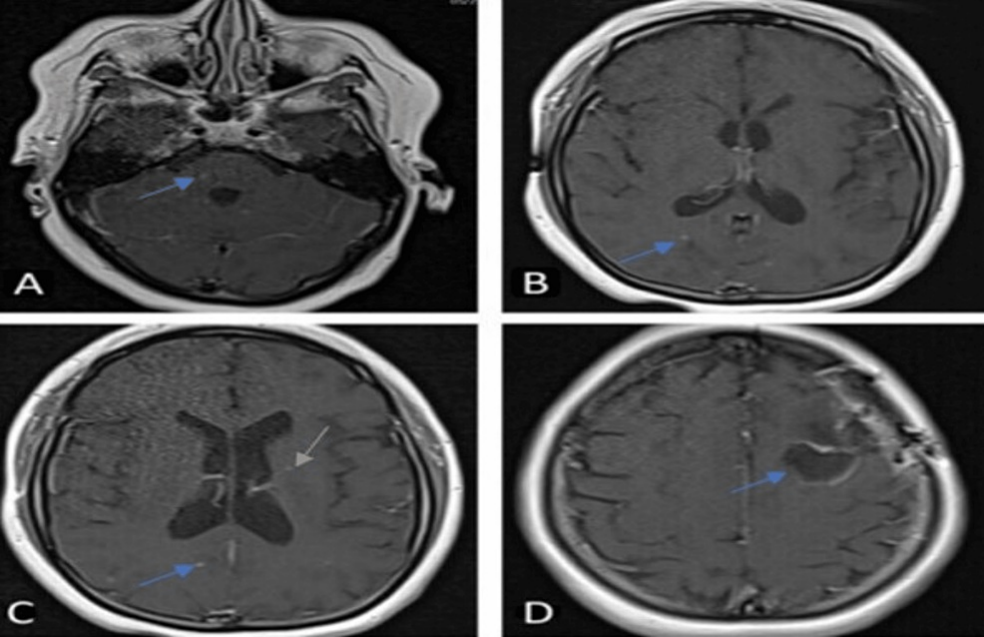
Brain MRI with contrast five weeks after discharge T1 with contrast axial sequence showing resolution of most lesion appreciated in Figure 1. (A) A small round enhancing lesion in the right medial pons (Blue arrow). (B) A small round enhancing lesion in the right subcortical occipital area (Blue arrow). (C) A small round enhancing lesion in the right subcortical occipital area (Blue arrow) and left corona radiata (orange arrow). (D) Expected progression of hematoma in the left frontal lobe (blue arrow).

The patient underwent a left frontal lobe biopsy for a definitive diagnosis, which was complicated by an intracerebral hemorrhage. PCR of the biopsy specimen confirmed Bartonella henselae infection. She was started on doxycycline and rifampin with prophylactic Trimethoprim/sulfamethoxazole (TMP-SMX). The patient was discharged home after one month with minimal improvement in vision. Brain MRI at five weeks post-discharge showed decreased number and size of lesions (Figures 2A-2D).

**Figure 3:**
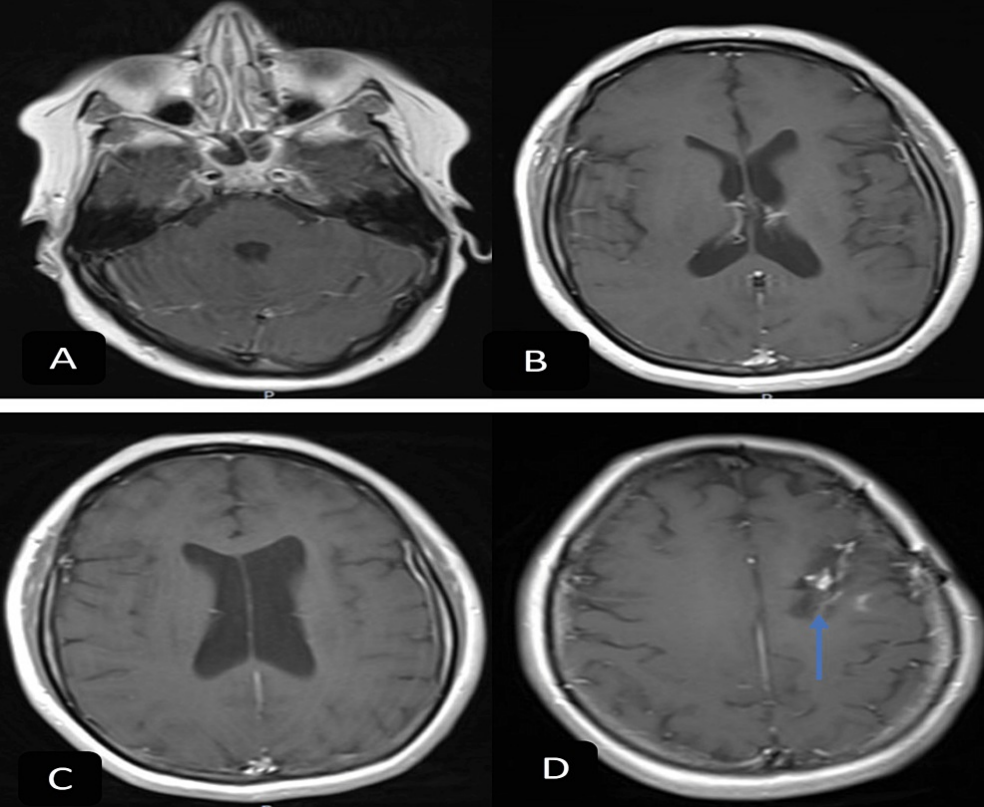
Brain MRI with contrast eight months after discharge T1 with contrast axial sequence showing resolution of multiple punctate enhancing lesions seen previously throughout the supratentorial and infratentorial regions (A-D). Expected progression of left frontal lobe hematoma (D, blue arrow).

She had a follow-up appointment with an infectious disease where a new brain MRI at eight months post-discharge was obtained and showed resolution of lesions (Figures 3A-3D). They decided to stop rifampin and continue doxycycline until further follow-up.

## Discussion

CNS involvement of Bartonella henselae is rare; meningitis, encephalitis, and neuroretinitis are the most common presentations [3-6]. According to our literature review, this is the first case of CNS Bartonellosis manifesting with multiple brain abscesses. We hypothesize that a profound degree of immunosuppression is required for this clinical presentation as we came across two cases of focal abscess caused by Bartonella where patients were immunocompetent [7,8]. Our patient received high doses of prednisone for a prolonged period in addition to mycophenolate mofetil.

Given Bartonella’s ability to infect erythrocytes, we believe that our patient’s infection disseminated hematogenously [7]. However, there is no clear explanation as to why it was localized to the CNS. This was also seen in another case of Bartonella brain abscess [8]. Perhaps, Bartonella has a tropism to the CNS vasculature we have not discovered yet. Diagnosing Bartonella henselae infection requires a combination of clinical suspicion and immunologic parameters [4]. Enzyme immunoassay to detect IgM and IgG antibodies is commonly used but PCR is the gold standard [8].

Bartonella CNS infection does not have a specific pattern on MRI. This is the first case of CNS Bartonellosis with multifocal ring-enhancing lesions in the brain. This is crucial, for it establishes Bartonella as a differential for infectious causes of multifocal ring-enhancing lesions in the brain such as Toxoplasma gondii, Mycobacterium tuberculosis, and Cryptococcus neoformans.

Regarding treatment, doxycycline, rifampin, macrolides, gentamicin, and chloramphenicol are among the most frequently selected antibiotics [7]. The regimen of oral doxycycline 100 mg BID and rifampin 300 mg BID was chosen for our patient. There is no evidence in regards to the duration of treatment; however, after eight months, our patient had a significant clinical improvement and radiologic resolution of lesions was seen on the MRI at follow-up, indicating treatment success.

## Conclusions

This case report aims to emphasize the consideration of CNS Bartonella henselae infection as an etiology of multiple brain lesions in immunocompromised patients. In our patient, brain MRI findings mimicked toxoplasmosis and neurocysticercosis. We encourage urgent evaluation for Bartonella if the standard workup for CNS infection is negative and there is no improvement with empiric treatment. Prompt diagnosis is of utmost importance as appropriate treatment is curative.
